# First person – Kaitlyn Rutter

**DOI:** 10.1242/dmm.052635

**Published:** 2025-09-17

## Abstract

First Person is a series of interviews with the first authors of a selection of papers published in Disease Models & Mechanisms, helping researchers promote themselves alongside their papers. Kaitlyn Rutter is first author on ‘ Retinopathy-associated inosine monophosphate dehydrogenase 1 mutations cause metabolic and filament defects in cones’, published in DMM. Kaitlyn is a postdoctoral scholar in the lab of Professor Susan Brockerhoff at University of Washington, Seattle, WA, USA, investigating how purines are synthesized in the retina in healthy and diseased states.


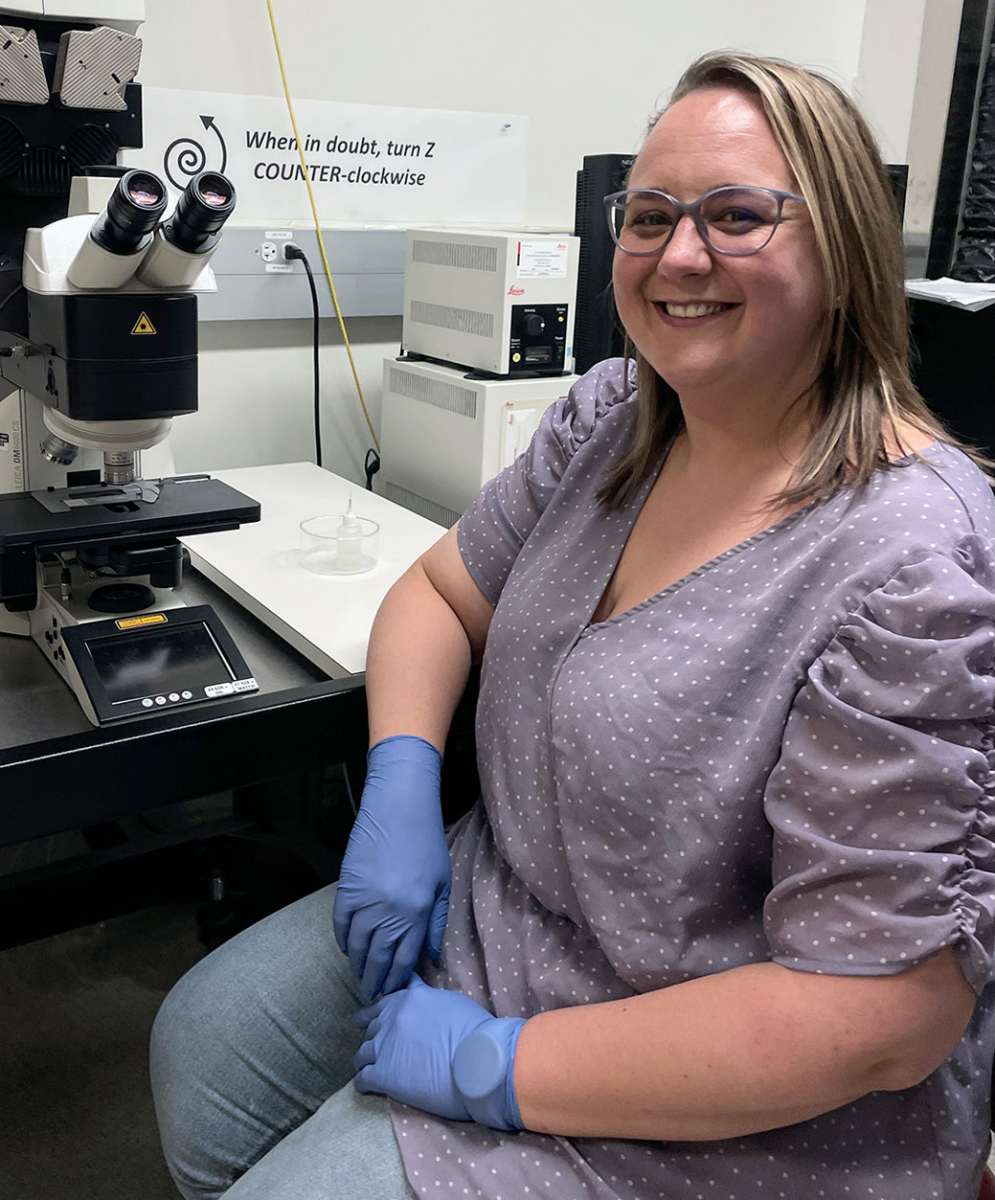


**Kaitlyn Rutter**

**Who or what inspired you to become a scientist?**

Growing up in a small town, I didn't have much exposure to science. During my first year of undergraduate education, I took a chemistry course that I thoroughly enjoyed. My professor, Dr Tim D'Andrea, instilled a level of confidence in me and inspired me to pursue a degree in science. From there, I joined Dr David Weinberg's inorganic chemistry research group, which further solidified my interest in science. To explore biochemistry research, I did a summer research project at a larger university in Dr Michael Cascio's lab. I was exposed to a glimpse of graduate school that summer and decided to pursue that path for myself. Though my research interests started off broad, I specifically felt drawn to conducting research that investigated disease mechanisms. This pull towards this type of research is connected to my grandmother's death from cancer – she has had a massive influence on my life, and I often felt helpless during the progression of the disease. As a scientist, I know that I can contribute to the larger field of continuously pushing knowledge forward to fight against disease and help prolong quality of life.

As a scientist, I know that I can contribute to the larger field of continuously pushing knowledge forward to fight against disease and help prolong quality of life

**What is the main question or challenge in disease biology you are addressing in this paper? How did you go about investigating your question or challenge?**

In this paper, we investigated whether mutations in inosine monophosphate dehydrogenase 1 (IMPDH1), which cause an autosomal dominant genetic disease leading to photoreceptor degeneration and blindness, result in hyperactivity of IMPDH1 *in vivo*. While *in vitro* experiments suggested these disease mutations might increase IMPDH1 activity and contribute to elevated cGMP levels – a factor linked to photoreceptor degeneration – we created the first animal models to examine this hypothesis directly in retinal tissue. Our main finding is that we found no evidence of IMPDH1 hyperactivity *in vivo* in the retina. However, we discovered that mutant IMPDH1 formed abnormally large and mislocalized filaments within the retina, highlighting a novel mechanism that could alter interactions with other proteins and impact cellular metabolism. This opens new directions for future research into the disease mechanism.

**How would you explain the main findings of your paper to non-scientific family and friends?**

Patients with a mutation in a protein involved in metabolism go blind, and we do not understand why. To develop therapies that prevent blindness, it is helpful to understand how and why cells in the eyes die. We made two zebrafish models that have the same mutations that human patients who are going blind do. The field had an interesting hypothesis that we wanted to test using our fish. The hypothesis was that this protein was active when it should not be, and this was causing a metabolite to build up in cells, which had previously been known to cause cell death. Although a very attractive hypothesis, we did not see any evidence of the buildup of that metabolite in the eye. In fact, in one of our fish models, we have evidence that the protein is less active, which is the opposite of what we expected. We also wanted to look at other hypotheses outside of metabolism, so we examined how this protein appeared under the microscope. Normally, it forms small filaments, but with these mutations, it formed massive filaments that accumulate in a specific area of the cell. Having big filaments in the wrong location could be detrimental to the cell and cause cell death.

**What are the potential implications of these results for disease biology and the possible impact on patients?**

One of the leading hypotheses for why patients with IMPDH1 mutations were going blind was that the enzyme was hyperactive. A plausible treatment would be to use an IMPDH1 inhibitor to prevent or delay disease progression. We generated the first IMPDH1 animal models to test this hypothesis in the context of the retina. In one of our models, we found that IMPDH1 was less active, which is opposite to what was proposed. In this case, if patients were treated with an IMPDH1 inhibitor, it could have made symptoms and disease progression worse. We used our zebrafish models to test other ideas, such as filament size and location. We can get more clues to why photoreceptors are degenerating in our models to eventually develop therapeutics that can prevent or delay blindness in humans.

We can get more clues to why photoreceptors are degenerating in our models to eventually develop therapeutics that can prevent or delay blindness in humans

**Figure:**
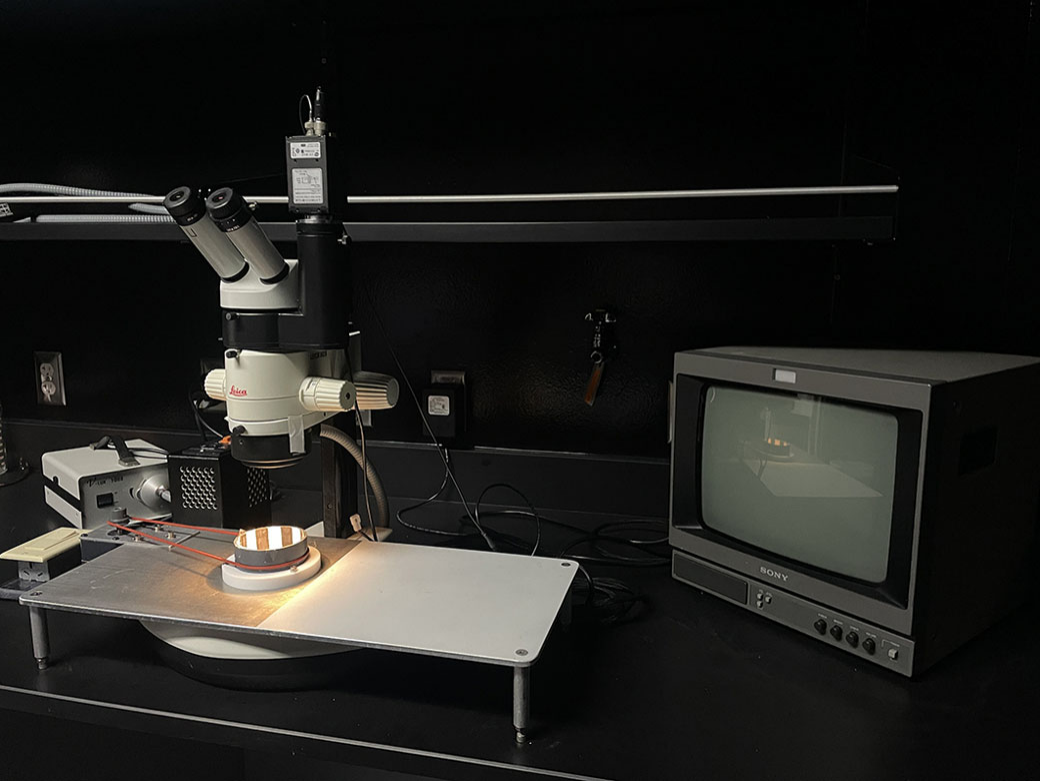
Optokinetic response apparatus used to assess zebrafish larval eye movements in response to rotating black and white stripes.

**Why did you choose DMM for your paper?**

I chose DMM because of the relevance to our work. We developed a novel zebrafish model for IMPDH1-related photoreceptor degeneration and tested hypotheses to inform us on the disease mechanism. DMM is an impactful journal that gives us an opportunity to share our work with a wide audience. The open access factor of DMM is also important for me. I believe in reducing barriers to people learning about the latest science – whether that be other scientists forming hypotheses for IMPDH1-related photoreceptor death, scientists studying less related work, patients or families struggling with this disease, or the general public eager to learn science.

**Given your current role, what challenges do you face and what changes could improve the professional lives of other scientists in this role?**

The most significant challenge that I've seen throughout my professional career is the stress of securing funding. I think there are several ways to help address this issue. As a graduate student, I was fortunate to be awarded an F31 grant from the National Institutes of Health. I feel I was successful in part due to the incredible mentorship I had – both from my direct PI and from a grant-writing course where another PI and peers read through my application. This was critical as some people were outside my field and asked probing questions that made me think about my work more deeply which I believe strengthened my application tremendously. Challenges persist however – such as the publish or perish mentality and the uncertainty of US government funding. I would advocate holding great significance to papers with either exciting new results or ones that disprove a hypothesis or have negative results. I also advocate for communicating science to the general public to help raise awareness for the importance of scientific funding. I believe that everyone is capable of learning about our work, and it is important for scientists to learn communication skills to convey complicated data to non-scientists.

**What's next for you?**

I am currently focused on expanding the studies in this paper. Now that we have these zebrafish models, I can use them to further define why cones are dying. I am also very interested in determining how purines are synthesized in the retina in both healthy and diseased states. Two pathways can synthesize purines – *de novo* purine synthesis and the salvage pathway. It is currently unknown what the contributions of those pathways are in the retina and if that balance shifts in the context of disease.

**Tell us something interesting about yourself that wouldn't be on your CV**

Outside of the lab, I enjoy both being out in nature and crafting indoors. I especially love being on the water and have since I was a little girl. In graduate school, I started kayaking and found it was restorative. During the gloomier months when being outside isn't as accessible, I enjoy painting ceramics, cross-stitching and other crafts to keep my hands busy.
